# Reduced Immune Fitness and Job Performance: Absenteeism, Presenteeism, and Associated Costs for the Dutch Economy

**DOI:** 10.3390/ijerph20031761

**Published:** 2023-01-18

**Authors:** Annabel S. M. Sips, Noortje R. Severeijns, Aletta D. Kraneveld, Johan Garssen, Joris C. Verster

**Affiliations:** 1 Division of Pharmacology, Utrecht Institute for Pharmaceutical Sciences, Utrecht University, 3584 CG Utrecht, The Netherlands; 2 Global Centre of Excellence Immunology, Nutricia Danone Research, 3584 CT Utrecht, The Netherlands; 3 Centre for Human Psychopharmacology, Swinburne University, Melbourne, VIC 3122, Australia

**Keywords:** immune fitness, absenteeism, presenteeism, work performance, impairment

## Abstract

Reduced immune fitness can have a significant negative impact on work performance. The aim of the current study was to evaluate the impact of reduced immune fitness on job performance and associated costs for the Dutch economy. Data from *n* = 425 Dutch working adults (18–65 years old) who completed an online survey were analyzed to evaluate the number of days of absenteeism (not going to work) and presenteeism (working while sick) due to reduced immune fitness, and the performance level on days worked with reduced immune fitness. Data from for the year 2019 were analyzed. Participants reported 2.9 absenteeism days and 19 presenteeism days, with an average performance reduction of 22.8% when working on days with reduced immune fitness. Significantly more days of absenteeism and presenteeism were reported by women, individuals with a poorer immune fitness, and those with underlying disease. Performance impairment on days worked while experiencing reduced immune fitness was significantly greater among individuals with a younger age at the junior career level, those with underlying disease, and among highly educated individuals. The associated costs of reduced immune fitness were estimated at €4.3 billion for absenteeism and €6.4 billion for presenteeism. Together, the costs of reduced immune fitness for the Dutch economy in 2019 were estimated at €10.7 billion. These findings demonstrate that reduced immune fitness has a significant negative impact on the Dutch economy.

## 1. Introduction

Immune fitness refers to the capacity of the body to respond to health challenges (such as infections) by activating an appropriate immune response, essential to maintain health, prevent and resolve disease, and improve quality of life [1]. Reduced immune fitness and experiencing disease can be possible reasons for absenteeism, i.e., not being able to work. Reduced immune fitness can also be the cause of presenteeism, i.e., working while sick leading to a decrease in productivity [2,3,4]. Reduced immune fitness is both a cause and consequence of having immune-related chronic diseases, such as cardiovascular disease, diabetes, depression, and asthma [5,6]. In 2021, 51.3% of the Dutch population had at least one or more of these chronic physical or mental health conditions [7]. In addition, otherwise healthy individuals may also experience reduced immune fitness, as they occasionally may experience a common cold and influenza [8]. Given this, reduced immune fitness is a common phenomenon affecting the general population.

The economic costs of reduced immune fitness due to chronic diseases should not be underestimated. For example, in 2017 the total estimated costs for the US economy of diabetes were $327 billion. Of these costs, $237 billion were direct medical costs (72%) and $90 billion was caused by reduced work productivity (presenteeism, 28%) [9]. An international study [10] revealed that the yearly costs of depression-related absenteeism per person in the US ($390) was lower compared to Brazil ($1361), Canada ($1567), Japan ($2674), Mexico ($928) and South Africa ($894), but higher than South Korea ($181) and China ($136). Regarding depression-related presenteeism, the yearly costs per person in the US ($5524) were lower than Brazil ($5788) and South Africa ($6066), but higher than Canada ($4270), China ($547), Japan ($3801), South Korea ($2114), and Mexico ($2918) [10]. In The Netherlands, studies also indicate that diseases related to reduced immune fitness cause absenteeism and presenteeism. For example, a Dutch study estimated the yearly costs of absenteeism and presenteeism among patients with rheumatoid arthritis at €6240 and €15,548 per person, respectively [11]. Together, these studies illustrate and underline the impact of immune-related disease on the economy. However, the findings also show that there are clear differences between countries. These differences may be related to variances in (access to) health care of the countries, but also differences between the countries in (or the absence of) legislation concerning sick leaves may play a role. 

While the cost of absenteeism can be easily measured (e.g., by counting sick days), presenteeism comprises costs that are less easily uncovered. As such, the costs of presenteeism often remain unmeasured and employers are not always aware of these costs [12,13]. Presenteeism could be measured by looking at reduced output of employees, failure to uphold the regular work standard, mistakes in work and other outputs [12]. Ignoring the costs of presenteeism will cause a significant underestimation of the real cost of reduced immune fitness for the employer [14]. In fact, research has shown that the costs of presenteeism exceed the costs of absenteeism [15]. For example, in the US study by Goetzel et al. [16], presenteeism accounted for 61% of the total economic costs for the employer, and in a Dutch study among patients with rheumatoid arthritis, presenteeism accounted for 71% of the total costs [11]. A US study among *n* = 5369 employees reported that 65% of them had one or more chronic medical conditions [17]. Depending on the condition, these employees reported an average of 0.9 to 5.9 h of absenteeism per week, and their self-reported productivity was reduced to 82.2% asthma, 76.2% (other breathing disorders), and 64.6% (emotional disorders) compared to performance levels of healthy employees [17]. A third US study among *n* = 16,651 employees found that 47% of the sample indicated to have one or more chronic conditions. Of this group, 21.7% reported impairments in physical work and 31.9% had reported limitations of their general work output [13]. Taken together, chronic health conditions are associated with significantly elevated levels of both absenteeism and presenteeism.

It is important to underline the fact that the individuals that participated in these studies discussed here were all experiencing underlying diseases. They do not represent the general healthy population that may experience more common immune-related complaints that result in reduced immune fitness such as common cold and influenza. In addition, there are various factors not related to medical conditions that also can influence absenteeism and presenteeism. For example, sex, age, and education level may influence health and disease, and thus immune fitness. Therefore, rates of presenteeism and absenteeism may differ between subgroups of the general population. Regarding sex, studies from Canada and UK found that women report higher absenteeism rates than men [18,19]. In a Dutch study, the difference in absenteeism days between men (9.9 days) and women (13.0 days) did not reach statistical significance [20]. Regarding presenteeism, Swedish studies found that women attended work sick more often than men [21,22]. However, these findings were not confirmed by another Swedish study [23]. Regarding age, it is known that older individuals more often have chronic health conditions, which may be reflected in more sick leave and reduced work productivity [24]. In general, with increasing age, the duration of absenteeism periods increases, as well as the probability of presenteeism [25]. For the European Union (EU) it was estimated that, while aging, the average duration of absenteeism of employees increases by 0.1 days per year [26]. Regarding education level, it has been reported that levels of absenteeism and presenteeism were lower among higher educated employees [10,27]. Finally, there may be several other factors influencing the frequency and duration of absenteeism and presenteeism, including, but not limited to, job role, work characteristics, work satisfaction, work-related stress, loyalty, and engagement [28].

The aim of the current investigation was to evaluate the impact of reduced immune fitness on absenteeism and presenteeism in a working population sample of The Netherlands. The analysis investigated the percentage of performance reduction on working days with reduced immune fitness and evaluate factors that may possibly influence absenteeism and presenteeism, including sex, age, education level, and job type. In addition, a rough estimate was made of the associated costs for the Dutch economy. Based on previous research, it was hypothesized that reduced immune fitness is associated with reduced productivity at work (presenteeism), as an increased number of days absent from work (absenteeism). These effects were hypothesized to be greater in women [21,22], individuals of older age [25], and those with a lower level of education [10,27].

## 2. Methods

Data for 2019 (pre-COVID-19) from the ‘Corona lockdown: how fit are you?’ (CLOFIT) study were used for the current analysis [29]. An online survey was conducted between the 24 June and the 26 July 2020. Dutch adults aged 18 years and older could participate in the study, and they were recruited through Facebook advertising. The Ethics Committee of the Faculty of Social and Behavioural Sciences of Utrecht University granted ethical approval (approval code: FETC17-061), and electronic informed consent was obtained from all participants at the start of the survey. Only participants who reported having a job in 2019 were included. A detailed description of the CLOFIT survey methodology and content can be found elsewhere [29]. For the current analysis, selected demographics, data on absenteeism and presenteeism related to reduced immune fitness, and assessments of immune fitness for the year 2019 were considered.

### 2.1. Demographics

Demographic data included sex and age. Individuals indicated their highest level of education from a listing of education types composed by Statistics Netherlands [30]. The education types were then recoded into education levels: (1) low, (2) middle, or (3) high education. Participants indicated their job status in 2019 by choosing among being employed, unemployed, student with a part-time job, or retired. Participants could indicate their job type according to the job categorization of Statistics Netherlands [31], based on the International Standard Classification of Occupations (ISCO 2008).

### 2.2. Absenteeism and Presenteeism Associated with Reduced Immune Fitness

Absenteeism and presenteeism related to reduced immune fitness were assessed for 2019. Questions were adapted from a study examining the costs of workplace hangovers and intoxication to the UK economy [32]. Questions concerned the number of days in 2019 that participants (a) did not work because they experienced reduced immune fitness (absenteeism) and (b) did work although they experienced reduced immune fitness (presenteeism). Regarding presenteeism, they could further indicate, in comparison to a regular working day without reduced immune fitness, how well they performed at work on days when they experienced reduced immune fitness. The performance level was rated on a scale ranging from 0% (‘compared to a regular day I achieved nothing/did not work’) to 100% (‘my work was absolutely not influenced by experiencing reduced immune fitness’).

### 2.3. Immune Fitness and Underlying Diseases

Immune fitness in 2019 was assessed with the Immune Status Questionnaire (ISQ) and a single item immune fitness scale [33]. The ISQ comprises of seven items that assess the frequency of occurrence of immune-related complaints including common cold, diarrhea, sudden high fever, headache, muscle and joint pain, skin problems (e.g., acne and eczema), and coughing [8]. Answering possibilities were never, sometimes, regularly, often, and (almost) always. The sum score of items is then recoded into a score ranging from very poor (0) to excellent (10) [8]. Participants could also rate their immune fitness for 2019 on a single item scale ranging from very poor (0) to excellent (10) [33,34].

Participants could indicate whether they had an underlying disease (chronic health conditions such as cardiovascular disease or hypertension, diabetes, liver disease, neurological diseases, immune disorders, allergy, kidney disease, pulmonary diseases, anxiety, depression, and sleep disorders), based on a listing by the Dutch National Institute for Public Health and the Environment (RIVM) [7].

### 2.4. Statistical Analysis

A total of *n* = 1910 individuals completed the CLOFIT study. However, only part of them had a job (part-time or fulltime) in 2019 and were included in the dataset for the current analysis (*n* = 489). The survey question on age was completed in 2020, whereas the cost estimate calculations comprise the year 2019. Therefore, to include only participants 18–65 years old, participants aged below 19 and 67 and above were excluded (*n* = 24). Data of *n* = 40 participants were judged unreliable and excluded because, despite reporting excellent immune fitness for 2019, (single item scores ≥ 8) they reported ≥50 days of absenteeism or presenteeism due to reduced immune fitness. The final sample for analysis included *n* = 425 working individuals.

**Figure 1:**
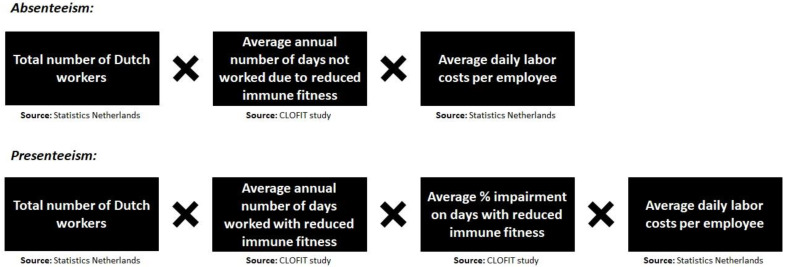
Formulas for calculation the economic costs of absenteeism and presenteeism due to reduced immune fitness.

Statistical analyses were conducted with SPSS (IBM Corp. Released 2013. IBM SPSS Statistics for Mac, Version 27.0. Armonk, NY, USA: IBM Corp.). Means and standard deviation (SD) were calculated, and distribution of the means were checked for normality. Since most data were not normally distributed, non-parametric tests were applied for the analysis. Percentual differences were evaluated with chi-squared tests. Differences in means between groups were evaluated with the Independent–Samples Mann–Whitney U–test (2 groups, e.g., sex) or the Independent–Samples Kruskal–Wallis test, applying a Bonferroni’s correction for multiple comparisons (more than 2 groups, e.g., education level). Spearman’s correlations were computed between the number of days of absenteeism and presenteeism, performance level, and the ISQ and the single item immune fitness score. Finally, the economic cost of absenteeism and presenteeism due to reduced immune fitness were estimated, applying the methodology used by Bhattacharya [32]. The calculation of the economic costs for absenteeism and presenteeism are summarized in Figure 1.

Information on the total number of Dutch workers in 2019 [35] and the average daily labor costs per employee were obtained from Statistics Netherlands [36]. The number of employees in 2019 between the age of 15 and 65 was 8,886,000 [35]. A correction based on the number of employees 15–20 years old (692,000) [35] to estimate the number of employees 18–65 years old, resulted in 8,886,000 − (3/6 × 692,000) = 8,540,000 employees. The average income (before VAT) for 2018 was €44,000 [36]. Corrected for inflation (2.6%) [37], the average yearly income for 2019 was estimated at €45.144 (€868.15 per week). The average hourly income in 2019 was calculated by dividing the average weekly income in 2019 (€868.15) by the average number of working hours per week (31 h) [38] and equaled €28. The average number of hours worked per working day was 31 h/5 working days = 6.2 h. Thus, the average daily labor cost was estimated at €28 × 6.2 h = €173.6 per day. Total costs of reduced immune fitness for the Dutch economy were estimated by calculating the sum of costs due to absenteeism and presenteeism.

## 3. Results

The final sample consisted of *n* = 425 subjects with a mean (SD) age of 36.2 (14.9) years old. A total 67.1% of the sample were women. Table 1 summarizes their demographics.

On average, participants worked 28.7 h per week divided over 4.3 working days. Higher educated participants at the junior career level were overrepresented in the sample. Several sex differences were observed. Men were significantly older than women and worked more often at the senior and less often at the junior career level. Men were overrepresented in the job categories industry, wholesale and retail trade, repair of cars, and transportation and storage, and underrepresented in health and welfare jobs. Men worked significantly more hours and days per week than women. No significant sex differences were found regarding education level or underlying disease.

Immune fitness and outcomes on absenteeism, presenteeism, and work performance are summarized in Table 2. Overall, 2.9 absenteeism days and 19 presenteeism days were reported in 2019, with a performance reduction of 22.8% on working days with reduced immune fitness.

### 3.1. Sex

The immune fitness scores on both ISQ and the single item rating were significantly lower in women compared to men (see Table 2). Women reported significantly more days of absenteeism and days of presenteeism than men. However, no significant sex difference was found regarding the performance level on working days with reduced immune fitness.

### 3.2. Immune Fitness

Table 3 summarizes the correlations between immune fitness, absenteeism, presenteeism, and performance on working days with reduced immune fitness.

Significant negative correlations were found between ISQ scores or immune fitness score, and days of absenteeism and presenteeism. ISQ and immune fitness score correlated significantly with each other (r = 0.293, *p* < 0.001). Performance levels on days with reduced immune fitness correlated significantly and positively with the ISQ, suggesting that experiencing less immune-related complaints is associated with less performance impairment on days with reduced immune fitness. However, the single item immune fitness score did not correlate significantly with performance impairment on days with reduced immune fitness.

### 3.3. Career Level and Age

The outcomes according to career level, i.e., a proxy measure of age, are summarized in Table 4. The analysis revealed that ISQ scores of individuals at the junior career level were significantly lower than those at the middle (*p* = 0.002) and senior career level (*p* < 0.001). The difference in experiencing immune-related complaints was not reflected in significant differences in immune fitness between the career levels (*p* = 0.167). Also, no significant differences according career level were found for days of absenteeism (*p* = 0.221) and days of presenteeism (*p* = 0.506). However, performance impairment on days worked with reduced immune fitness was significantly greater among individuals at the junior career level (−25.7%) compared to individuals at the middle career level (−16.6%, *p* < 0.001) and the senior career level (−20.0%, *p* < 0.001).

A significant positive correlation was found between age and performance level on days with reduced immune fitness (r = 0.207, *p* < 0.001), suggesting that with increasing age, reduced immune fitness has less impact on work performance. Age did not significantly correlate with days of absenteeism (r = −0.078, *p* = 0.107) or presenteeism (r = −0.050, *p* = 0.301).

### 3.4. Education Level

The outcomes according to education level are summarized in Table 5. Although immune fitness scores were better with higher education levels, this effect was not statistically significant. Also, no statistical differences according to education level were found for days of absenteeism and days of presenteeism. However, performance impairment on working days with reduced immune fitness was significantly greater among high educated individuals (−25.7%) compared to individuals with middle level education (−18.4%, *p* < 0.001) and a low education level (−16.5%, *p* = 0.002).

### 3.5. Underlying Disease

The outcomes according to underlying disease status are summarized in Table 6. Individuals with an underlying disease had a significantly lower score on the ISQ (*p* < 0.001) and reported significantly poorer immune fitness (*p* < 0.001) compared to individuals without underlying disease. Individuals with an underlying disease further reported significantly more days of absenteeism (*p* < 0.001), days of presenteeism (*p* < 0.001), and a greater reduction in performance level when working on days with reduced immune fitness (−24.9%, *p* = 0.038) compared to individuals without underlying diseases (−20.3%).

### 3.6. Estimated Costs

**Figure 2:**
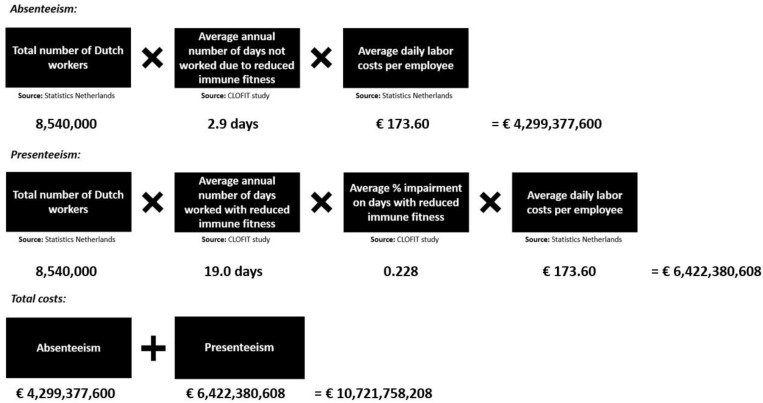
Estimated costs of reduced immune fitness for the Dutch economy in 2019.

The estimated costs of absenteeism and presenteeism due to reduced immune fitness were estimated by applying the methodology used by Bhattacharya [32]. The calculations are summarized in Figure 2. The costs of absenteeism were estimated at €4,299,377,600 and the costs of presenteeism at €6,422,380,600. Together, the costs of reduced immune fitness for the Dutch economy in 2019 were estimated at €10,721,758,208.

## 4. Discussion

In 2019, reduced immune fitness was associated with an average of 2.9 absenteeism days and 19 presenteeism days, and a performance reduction of 22.8% when working on days with reduced immune fitness. Significantly more days of absenteeism and presenteeism were reported by women, individuals with a poorer immune fitness, and those with underlying disease. Performance impairment on days worked with reduced immune fitness was significantly greater among individuals with a younger age at the junior career level, those with underlying disease, and among high educated individuals. The associated costs for the Dutch economy were estimated at €4.3 billion for absenteeism and €6.4 billion for presenteeism. Together, the costs of reduced immune fitness for the Dutch economy in 2019 were estimated at €10.7 billion. The number of presenteeism days was more than 6 times higher than the number of absenteeism days. The estimated associated costs for the Dutch economy of presenteeism were more than two times higher than those of absenteeism. The total estimated costs of reduced immune fitness of €10.7 billion correspond to €1255 per employee for 2019. Together, these findings suggest that reduced immune fitness has a significant negative impact on the Dutch economy. Implementing strategies to improve and maintain adequate immune fitness are therefore warranted.

The findings are in line with previous research showing that the costs of immune-related chronic diseases for the economy are high [9,10,11]. The observation that performance levels were reduced to 82.2–64.6% of normal productivity levels in individuals with underlying diseases [17] are in line with the performance level of 75.1% reported for working on days with reduced immune fitness for those with underlying disease in the current study. The observation that women reported significantly more days of absenteeism and presenteeism is also in line with previous studies and our hypothesis [18,19,20,21,22]. Regarding age, previous research showed that older individuals more often have chronic health conditions, which was associated with reduced work productivity in older adults [24], and more days of absenteeism and presenteeism [25]. The current study did not confirm these findings: no significant difference in days of absenteeism and presenteeism was found between junior, middle, and senior workers. However, the youngest age group (early career) reported a significant greater impact of reduced immune fitness on work performance level on presenteeism days compared to the middle and senior career groups. Finally, previous research found that levels of absenteeism and presenteeism were lower among higher educated employees [10,27]. In the current study no differences were found according to education level. Contrary to these findings, it was found that performance impairment on days worked with reduced immune fitness was greatest among individuals with a high education level. Taken together, our hypothesis that reduced immune fitness is associated with reduced productivity at work, increased absenteeism and presenteeism, and that these effects were greater in women than men were confirmed. However, no significant effects of age and education level were found.

The current study had several limitations which should be considered when interpreting the presented data. The limitations discussed in this paragraph may have minor impact on the reported performance levels when working with reduced immune fitness. However, they may have impacted the reported number of days of absenteeism and presenteeism, and thus the estimation of associated costs for the Dutch economy. First, the study comprised a convenience sample. For several aspects the sample is therefore not representative for the Dutch adult working population. For example, not all job categories are represented according to the known Dutch distribution, and women and participants with a higher education level were overrepresented. Second, it was not feasible to include actual labor costs for each individual, or the average costs according to job category. Instead, as an estimate the average labor costs of a Dutch employee was used for the calculations. Third, as the data was collected retrospectively in June and July 2020 for the year 2019 it is possible that the results are influenced by recall bias. Also, the COVID-19 pandemic may have influenced their perception of health and viewpoints on immune fitness. Fourth, the study was conducted in The Netherlands. It is therefore unclear to what extent its outcomes can be generalized to other countries with different economies (e.g., low- and middle-income countries), different cultures, and different population demographics. It is therefore important that this study will be replicated in other countries. Finally, the sample size was relatively small. Therefore, no multivariate analyses were conducted to evaluate the influences of the independent variables (e.g., sex and education level) controlled by the influence of the other variables (e.g., job category) on reporting absenteeism, presenteeism, and reduced performance levels. Future studies should therefore comprise larger and more diverse samples to enable such analysis.

Given the limitations of the study, the associated costs of reduced immune fitness for the Dutch economy should be viewed as crude estimates only. Nevertheless, the estimated amount of €10.7 billion euro does indicate that the costs associated with reduced immune fitness are significant and relevant. As a comparison, in 2019 the Dutch government spent €9.5 billion on Infrastructure and Water Management, €10.0 billion on Foreign Affairs, €10.5 billion on Defense, and €12.7 billion on Justice and Security [39]. Future research is needed to more accurately estimate the actual costs of reduced immune fitness on the Dutch economy. Since the 2019 coronavirus disease (COVID-19) pandemic, several changes have been observed including working from home and flexible working hours that may impact absenteeism, presenteeism, and work performance level. Furthermore, health characteristics such as body mass index [40] and levels of pre-COVID-19 immune fitness [41] were shown to have an impact on changes in immune fitness and disease vulnerability during the pandemic. A new study can take these changes and health characteristics into account. The study should comprise a national representative sample of the Dutch working population with a representative distribution of sex, education level and job categories. Then, the actual labor costs should be used for the calculation instead of the average labor costs of a Dutch employee. It would then also be possible to calculate the cost of the absenteeism and presenteeism per job category. This is important as previous research found differences between job categories regarding absenteeism and presenteeism [21]. To achieve this, a larger sample size than the current study is needed. It would also be interesting to evaluate non-labor costs of reduced immune fitness, such as possible medical care and the use of OTC and prescription medication.

Finally, in future research it is also important to determine the major causes of reduced immune fitness. Are most of the economic costs related to reduced immune fitness due to underlying immune-related diseases, or have the more common immune-related complaints that are affecting the population as a whole the biggest impact on work absenteeism, presenteeism, and work performance? The answer to this question has significant consequences for policymakers. For example, should prevention work focus primarily on individuals with underlying disease (e.g., promote influenza vaccination), should policymakers aim to improve and maintain adequate immune fitness of the general population (e.g., promote regular exercise and a healthy diet), or both?

## 5. Conclusions

In conclusion, in 2019 (pre-COVID-19) reduced immune fitness was associated with an average of 2.9 absenteeism days and 19 presenteeism days, and a performance reduction of 22.8% on working days with reduced immune fitness. Absenteeism and presenteeism days were more frequently reported by women, individuals with a poorer immune fitness, and those with underlying disease. Performance impairment on days worked with reduced immune fitness was significantly greater among younger individuals (junior career level), those with underlying disease, and among higher educated individuals. The associated costs for the Dutch economy were estimated at €10.7 billion (€4.3 billion for absenteeism and €6.4 billion for presenteeism). Future studies should verify these estimates in a larger national representative sample.

## Figures and Tables

**Table 1:** Demographics.

Variables Assessed	Overall	Men	Women	*p*-Value
*n* (%)	425 (100)	140 (32.9)	285 (67.1)	<0.001 *
Age (Mean, SD)	36.2 (14.9)	39.4 (14.9)	34.6 (14.7)	0.001 *
Career level				
Junior (18–34 years old), *n* (%)	244 (57.4)	67 (47.9)	177 (62.1)	0.005 *
Middle (35–50 years old), *n* (%)	79 (18.6)	30 (21.4)	49 (17.2)	0.296
Senior (51–65 years old), *n* (%)	102 (24.0)	43 (30.7)	59 (20.7)	0.023 *
Education level, *n* (%)				
Low	49 (11.5)	15 (10.7)	34 (11.9)	0.716
Middle	112 (26.4)	38 (27.2)	74 (26.0)	0.792
High	264 (62.1)	87 (62.1)	177 (62.1)	1.000
Job category, *n* (%)				
Agriculture, forestry, fishing	7 (1.6)	3 (2.1)	4 (2.4)	0.847
Industry	19 (4.5)	12 (8.6)	7 (2.5)	0.004 *
Production and distribution of electricity, natural gas, steam and cooled air trade	3 (0.7)	1 (0.7)	2 (0.7)	1.000
Construction industry	6 (1.4)	2 (1.4)	4 (1.4)	1.000
Wholesale and retail trade, repair of cars	20 (4.7)	12 (8.6)	8 (2.8)	0.008 *
Transportation and storage	19 (4.5)	13 (9.3)	6 (2.1)	<0.001 *
Hospitality, catering industry	37 (8.7)	10 (7.1)	27 (9.5)	0.410
Information and communication	29 (6.8)	12 (8.6)	17 (6.0)	0.319
Financial services	18 (4.2)	9 (6.4)	9 (3.2)	0.125
Rental and trade of real estate	1 (0.2)	0 (0.0)	1 (0.4)	0.454
Advice, research and other specialist business services	23 (5.4)	6 (4.3)	17 (6.0)	0.468
Rental and trade of real estate	2 (0.5)	1 (0.7)	1 (0.4)	0.680
Public administration, government, social insurance	16 (3.8)	5 (3.6)	11 (3.9)	0.879
Education	41 (9.6)	13 (9.3)	28 (9.8)	0.870
Health and welfare care	105 (24.7)	20 (14.3)	85 (29.8)	<0.001 *
Culture, sports and recreation	25 (5.9)	5 (3.6)	20 (7.0)	0.162
Other services	51 (12.0)	16 (11.4)	35 (12.3)	0.789
Households as employers	3 (0.7)	0 (0.0)	3 (1.1)	0.214
Work characteristics, mean (SD)				
Hours worked per week	28.7 (13.5)	33.2 (14.5)	26.5 (12.4)	<0.001 *
Days worked per week	4.3 (1.8)	4.6 (2.0)	4.1 (1.6)	0.008 *
Days worked on location per week	3.7 (1.6)	4.0 (1.7)	3.6 (1.5)	0.006 *
Days worked from home per week	0.5 (1.3)	0.6 (1.5)	0.5 (1.3)	0.983
Underlying disease, *n* (%)				
Yes	230 (54.8)	67 (48.6)	163 (57.8)	0.074
No	190 (45.2)	71 (51.4)	119 (42.2)	0.074

Significant sex differences (*p* < 0.05) are indicated by *.

**Table 2:** Immune fitness, absenteeism, presenteeism, and work performance.

Variables Assessed	Overall	Men	Women	*p*-Value
Immune Fitness				
ISQ	7.0 (2.3)	7.7 (2.1)	6.7 (2.3)	<0.001 *
Single item immune fitness	7.6 (1.6)	7.9 (1.3)	7.4 (1.8)	0.008 *
Consequences of reduced immune fitness				
Absenteeism (days)	2.9 (9.3)	1.5 (3.3)	3.6 (11.0)	0.002 *
Presenteeism (days)	19.0 (56.1)	14.2 (48.9)	21.3 (59.2)	<0.001 *
Performance level with reduced immune fitness (%)	77.2 (17.7)	76.7 (19.0)	77.2 (17.2)	0.855

Significant sex differences (*p* < 0.05) are indicated by *. Abbreviation: ISQ = immune status questionnaire.

**Table 3:** Correlations between immune fitness (ISQ or single item immune fitness score), absenteeism, presenteeism, and work performance on days with reduced immune fitness.

Correlations	ISQ	Immune Fitness Score
Absenteeism (days)	r = −0.366, *p* < 0.001 *	r = −0.272, *p* < 0.001 *
Presenteeism (days)	r = −0.353, *p* < 0.001 *	r = −0.256, *p* < 0.001 *
Performance level (%) ^1^	r = 0.212, *p* < 0.001 *	r = 0.070, *p* = 0.243

Significant Spearman’s correlations (*p* < 0.05) are indicated by *. ^1^ = performance level on working days with reduced immune fitness. Abbreviation: ISQ = immune status questionnaire.

**Table 4:** Absenteeism, presenteeism, and work performance on days with reduced immune fitness according to career level.

Career Level	Junior	Middle	Senior
Age range (years)	18–35	36–50	51–65
*n*	244	79	102
ISQ	6.6 (2.1)	7.4 (2.3)	7.8 (2.3)
Immune fitness	7.7 (1.5)	7.5 (1.6)	7.3 (2.0)
Absenteeism (days)	2.4 (7.2)	1.9 (4.1)	4.6 (14.8)
Presenteeism (days)	11.9 (39.6)	36.8 (81.8)	22.1 (62.3)
Performance level (%) ^1^	74.3 (14.8)	83.4 (18.5) *	80.0 (22.9) *

Significant differences (*p* < 0.017, after Bonferroni’s correction for multiple comparisons) from the junior career level are indicated by *. ^1^ = performance level on days with reduced immune fitness. Abbreviation: ISQ = immune status questionnaire.

**Table 5:** Absenteeism, presenteeism, and work performance on days with reduced immune fitness according to education career level.

Education Level	Low	Middle	High
*n*	49	112	264
ISQ	7.3 (2.6)	7.1 (2.1)	7.0 (2.3)
Immune fitness	7.0 (2.2)	7.5 (1.7)	7.7 (1.4)
Absenteeism (days)	1.9 (4.6)	4.7 (14.1)	2.3 (6.9)
Presenteeism (days)	22.6 (59.0)	27.0 (70.2)	14.9 (48.0)
Performance level (%) ^1^	83.5 (18.7) *	81.6 (17.2) *	74.3 (17.2)

Significant differences (*p* < 0.017, after Bonferroni’s correction for multiple comparisons) compared to the high education level are indicated by *. ^1^ = performance level on days with reduced immune fitness. Abbreviation: ISQ = immune status questionnaire.

**Table 6:** Absenteeism, presenteeism, and work performance on days with reduced immune fitness according to underlying disease status.

Education Level	Underlying Disease	No Underlying Disease
*n*	230	190
ISQ	6.5 (2.3)	7.7 (2.0) *
Immune fitness	7.3 (1.7)	7.9 (1.4) *
Absenteeism (days)	3.8 (10.4)	1.8 (7.7) *
Presenteeism (days)	23.6 (62.7)	13.6 (47.3) *
Performance level (%) ^1^	75.1 (18.3)	79.7 (16.4) *

Significant differences (*p* < 0.05) from the high education level are indicated by *. ^1^ = performance level on days with reduced immune fitness. Abbreviation: ISQ = immune status questionnaire.

## Data Availability

The survey and data are available upon request from the corresponding author.

## References

[1] Van de Loo A.J.A.E., Kerssemakers N., Scholey A., Garssen J., Kraneveld A.D., Verster J.C. (2020). Perceived immune fitness, individual strength and hangover severity. Int. J. Environ. Res. Public Health.

[2] Schultz A.B., Chen C.Y., Edington D.W. (2009). The cost and impact of health conditions on presenteeism to employers. Pharmacoeconomics.

[3] Lofland J.H., Pizzi L., Frick K.D. (2004). A review of health-related workplace productivity loss instruments. Pharmacoeconomics.

[4] Prasad M., Wahlqvist P., Shikiar R., Shih Y.C.T. (2004). A review of self-report instruments measuring health-related work productivity. Pharmacoeconomics.

[5] Aggarwal B.B., Krishnan S., Guha S. (2012). Inflammation, Lifestyle and Chronic Diseases. The Silent Link.

[6] Furman D., Campisi J., Verdin E., Carrera-Bastos P., Targ S., Franceschi C., Ferrucci L., Gilroy D.W., Fasano A., Miller G.W. (2019). Chronic inflammation in the etiology of disease across the life span. Nat. Med..

[7] RIVM Aandoeningen. Welke Aandoeningen Hebben We in de Toekomst?. https://www.vtv2018.nl/aandoeningen.

[8] Wilod Versprille L.J.F., van de Loo A.J.A.E., Mackus M., Arnoldy L., Sulzer T.A.L., Vermeulen S.A., Abdulahad S., Huls H., Baars T., Kraneveld A.D. (2019). Development and validation of the Immune Status Questionnaire (ISQ). Int. J. Environ. Res. Public Health.

[9] American Diabetes Association (2018). Economic costs of diabetes in the US in 2017. Diabetes Care.

[10] Evans-Lacko S., Knapp M. (2016). Global patterns of workplace productivity for people with depression: Absenteeism and presenteeism costs across eight diverse countries. Soc. Psychiatry Psychiatr. Epidemiol..

[11] Braakman-Jansen L.M., Taal E., Kuper I.H., van de Laar M.A. (2012). Productivity loss due to absenteeism and presenteeism by different instruments in patients with RA and subjects without RA. Rheumatology.

[12] Burton W.N., Conti D.J., Chen C.Y., Schultz A.B., Edington D.W. (1999). The role of health risk factors and disease on worker productivity. J. Occup. Environ. Med..

[13] Burton W.N., Pransky G., Conti D.J., Chen C.Y., Edington D.W. (2004). The association of medical conditions and presenteeism. J. Occup. Environ. Med..

[14] Kigozi J., Jowett S., Lewis M., Barton P., Coast J. (2017). The estimation and inclusion of presenteeism costs in applied economic evaluation: A systematic review. Value Health.

[15] Mattke S., Balakrishnan A., Bergamo G., Newberry S.J. (2007). A review of methods to measure health-related productivity loss. Am. J. Manag. Care.

[16] Goetzel R.Z., Long S.R., Ozminkowski R.J., Hawkins K., Wang S., Lynch W. (2004). Health, absence, disability, and presenteeism cost estimates of certain physical and mental health conditions affecting US employers. J. Occup. Environ. Med..

[17] Collins J.J., Baase C.M., Sharda C.E., Ozminkowski R.J., Nicholson S., Billotti G.M., Turpin R.S., Olson M., Berger M.L. (2005). The assessment of chronic health conditions on work performance, absence, and total economic impact for employers. J. Occup. Environ. Med..

[18] Barmby T. (2002). Worker absenteeism: A discrete hazard model with bivariate heterogeneity. Labour Econ..

[19] Dionne G., Dostie B. (2007). New evidence on the determinants of absenteeism using linked employer-employee data. ILR Rev..

[20] Bekker M.H., Croon M.A., Bressers B. (2005). Childcare involvement, job characteristics, gender and work attitudes as predictors of emotional exhaustion and sickness absence. Work Stress.

[21] Aronsson G., Gustafsson K., Dallner M. (2000). Sick but yet at work. An empirical study of sickness presenteeism. J. Epidemiol. Community Health.

[22] Sendén M.G., Schenck-Gustafsson K., Fridner A. (2016). Gender differences in reasons for sickness presenteeism-a study among GPs in a Swedish health care organization. Ann. Occup. Environ. Med..

[23] Aronsson G., Gustafsson K. (2005). Sickness presenteeism: Prevalence, attendance-pressure factors, and an outline of a model for research. J. Occup. Environ. Med..

[24] Lerner D., Allaire S.H., Reisine S.T. (2005). Work disability resulting from chronic health conditions. J. Occup. Environ. Med..

[25] Bierla I., Huver B., Richard S. (2013). New evidence on absenteeism and presenteeism. Int. J. Hum. Resour. Manag..

[26] Frick B., Malo M.Á. (2008). Labor market institutions and individual absenteeism in the European Union: The relative importance of sickness benefit systems and employment protection legislation. Ind. Relat..

[27] Stewart W.F., Ricci J.A., Chee E., Morganstein D. (2003). Lost productive work time costs from health conditions in the United States: Results from the American Productivity Audit. J. Occup. Environ. Med..

[28] Prater T., Smith K. (2011). Underlying factors contributing to presenteeism and absenteeism. J. Bus. Econ. Res..

[29] Kiani P., Merlo A., Saeed H.M., Benson S., Bruce G., Hoorn R., Kraneveld A.D., Severeijns N.R., Sips A.S.M., Scholey A. (2021). Immune fitness, and the psychosocial and health consequences of the COVID-19 pandemic lockdown in The Netherlands: Methodology and design of the CLOFIT study. Eur. J. Investig. Health Psychol. Educ..

[30] Centraal Bureau voor de Statistiek (CBS) Opleidingsniveau. https://www.cbs.nl/nl-nl/nieuws/2019/33/verschil-levensverwachting-hoog-en-laagopgeleid-groeit/opleidingsniveau.

[31] Centraal Bureau voor de Statistiek (CBS) (2016). Uurlonen van Werknemers Naar Beroepsgroep. https://www.cbs.nl/nl-nl/maatwerk/2017/48/uurlonen-van-werknemers-naar-beroepsgroep-2016.

[32] Bhattacharya A. (2019). Financial Headache. The Cost of Workplace Hangovers and Intoxication to the UK Economy.

[33] Verster J.C., Kraneveld A.D., Garssen J. (2023). The assessment of immune fitness. J. Clin. Med..

[34] Van Schrojenstein Lantman M., Otten L.S., Mackus M., de Kruijff D., van de Loo A.J.A.E., Kraneveld A.D., Garssen J., Verster J.C. (2017). Mental resilience, perceived immune functioning, and health. J. Multidiscip. Healthc..

[35] Centraal Bureau voor de Statistiek (CBS) (2020). Arbeidsdeelname; Kerncijfers. https://opendata.cbs.nl/statline/?dl=6CBD5#/CBS/nl/dataset/85264NED/table.

[36] Centraal Bureau voor de Statistiek (CBS) Werkzame Beroepsbevolking; Gemiddeld Inkomen. https://opendata.cbs.nl/statline/#/CBS/nl/dataset/83686NED/table?dl=3451A.

[37] Media T. Inflatie Nederland 2019. https://www.inflation.eu/nl/inflatiecijfers/nederland/historische-inflatie/cpi-inflatie-nederland-2019.aspx.

[38] Centraal Bureau voor de Statistiek (CBS) Meer dan de Helft Werkt Voltijds. https://www.cbs.nl/nl-nl/nieuws/2020/08/meer-dan-de-helft-werkt-voltijds.

[39] Rijksoverheid Budget 2019. www.rijksfinancien.nl/begroting/uitgaven.

[40] Kiani P., Mulder K.E.W., Balikji J., Kraneveld A.D., Garssen J., Verster J.C. (2022). Pandemic preparedness: Maintaining adequate immune fitness by attaining a normal, healthy bodyweight. J. Clin. Med..

[41] Kiani P., Balikji J., Kraneveld A.D., Garssen J., Bruce G., Verster J.C. (2022). Pandemic preparedness: The importance of adequate immune fitness. J. Clin. Med..

